# Resting-State Functional Connectivity Is Associated With Cerebrospinal Fluid Levels of the Synaptic Protein NPTX2 in Non-demented Older Adults

**DOI:** 10.3389/fnagi.2019.00132

**Published:** 2019-06-07

**Authors:** Anja Soldan, Abhay Moghekar, Keenan A. Walker, Corinne Pettigrew, Xirui Hou, Hanzhang Lu, Michael I. Miller, Alfonso Alfini, Marilyn Albert, Desheng Xu, Mei-Fang Xiao, Paul Worley, The BIOCARD Research Team, John Csernansky

**Affiliations:** ^1^Department of Neurology, Johns Hopkins University School of Medicine, Baltimore, MD, United States; ^2^Department of Radiology, Johns Hopkins University School of Medicine, Baltimore, MD, United States; ^3^Department of Biomedical Engineering, Johns Hopkins University, Baltimore, MD, United States; ^4^Department of Mental Health, Johns Hopkins Bloomberg School of Public Health, Baltimore, MD, United States; ^5^Department of Neuroscience, Johns Hopkins University School of Medicine, Baltimore, MD, United States

**Keywords:** resting-state functional connectivity, magnetic resonance imaging, cerebrospinal fluid, NPTX2, synaptic function

## Abstract

Intrinsic functional connectivity of large-scale brain networks has been shown to change with aging and Alzheimer’s disease (AD). These alterations are thought to reflect changes in synaptic function, but the underlying biological mechanisms are poorly understood. This study examined whether Neuronal Pentraxin 2 (NPTX2), a synaptic protein that mediates homeostatic strengthening of inhibitory circuits to control cortical excitability, is associated with functional connectivity as measured by resting-state functional magnetic resonance imaging (rsfMRI) in five large-scale cognitive brain networks. In this cross-sectional study, rsfMRI scans were obtained from 130 older individuals (mean age = 69 years) with normal cognition (*N* = 113) and Mild Cognitive Impairment (*N* = 17); NPTX2 was measured in the same individuals in cerebrospinal fluid (CSF). Higher levels of NPTX2 in CSF were associated with greater functional connectivity in the salience/ventral attention network, based on linear regression analysis. Moreover, this association was stronger among individuals with lower levels of cognitive reserve, as measured by a composite score (comprised of years of education, reading, and vocabulary measures). Additionally, higher connectivity in the salience/ventral attention network was related to better performance on a composite measure of executive function. Levels of NPTX2 were not associated with connectivity in other networks (executive control, limbic, dorsal attention, and default-mode). Findings also confirmed prior reports that individuals with MCI have lower levels of NPTX2 compared to those with normal cognition. Taken together, the results suggest that NPTX2 mechanisms may play a central role among older individuals in connectivity within the salience/ventral attention network and for cognitive tasks that require modulation of attention and response selection.

## Introduction

It is increasingly recognized that aging and Alzheimer’s disease (AD) are accompanied by alterations in functional connectivity in large-scale brain networks, as measured by resting-state functional magnetic resonance imaging (rsfMRI) (e.g., Betzel et al., 2014; Dennis and Thompson, 2014; Badhwar et al., 2017). For example, a recent meta-analysis reported that functional connectivity as measured by rsfMRI differs among patients with AD dementia compared to those with normal cognition in three specific networks: the default-mode network, the salience/ventral attention network, and the limbic network (Badhwar et al., 2017). Functional connectivity changes in these networks also tend to be smaller and less consistent among patients with Mild Cognitive Impairment (MCI) (Badhwar et al., 2017). Additionally, age-related decreases in functional connectivity have been reported for the default-mode and salience/ventral attention networks (e.g., Sala-Llonch et al., 2015; Zonneveld et al., 2019).

Despite the increasing evidence for changes of rsfMRI connectivity in aging and AD, little is known about the biological mechanisms underlying alterations in these intrinsic brain networks. Activity measured by rsfMRI is generally considered to be related to fluctuations in dendritic potentials at excitatory synapses, often in the high frequency (gamma) band (e.g., Scholvinck et al., 2010; Hutchison et al., 2015). To reach fMRI detection threshold, a substantial population of neurons must be co-activated within a particular region, suggesting a requirement for synchrony. Covariance across brain areas is assumed to require long-range synaptic connectivity and recent models of long-range functional connectivity implicate a prominent role for interneurons that mediate local and long-range control of rhythmicity and synchrony (Stroud and Vogels, 2018).

NPTX2 is a synaptic protein that mediates adaptive strengthening of specific-interneuron circuits and is important for rhythmic synchrony (gamma power) of pyramidal neurons (Chang et al., 2010; Pelkey et al., 2015; Xiao et al., 2017). Recent evidence indicates that NPTX2 expression is reduced in brain tissue from patients with AD dementia compared to controls. Several lines of evidence indicate that NPTX2 expression and interneuron function are also reduced with aging, albeit to a lesser extent than in AD (Swanson et al., 2016; Xiao et al., 2017). Additionally, CSF levels of NPTX2 are reduced in patients with AD dementia and MCI, compared to controls and correlate with cognitive performance (Xiao et al., 2017).

The findings that both NPTX2 and functional connectivity measured by rsfMRI are linked to intrinsic brain rhythmicity and both change with aging and AD, suggests that levels of NPTX2 in CSF may be related to functional connectivity in large-scale brain networks. Therefore, the current study tested the hypothesis that lower levels of NPTX2 would be associated with lower functional connectivity in networks sensitive to aging and AD among non-demented older adults, particularly the default-mode network and the salience/ventral-attention network.

In addition, we examined whether important risk factors for AD moderate the relationship between NPTX2 and functional connectivity, including APOE-e4 genetic status, the main genetic risk for AD (Farrer et al., 1997), advancing age, and level of cognitive reserve (CR). CR was included in these analyses, as it is thought to be a property of the brain that confers “cognitive resilience,” allowing for sustained cognitive performance in the face of age or disease-related brain changes (for a review, see Stern et al., 2018; Pettigrew and Soldan, 2019). Because some studies have reported associations between measures of CR and measures of rsfMRI connectivity (e.g., Marques et al., 2016; Franzmeier et al., 2017a,b; Serra et al., 2017), we hypothesized that CR may moderate the association between NPTX2 and resting state connectivity. As a secondary goal, we also explored whether the association between NPTX2 and diagnostic status (i.e., cognitively normal, MCI) differed as a function of age, APOE e4 carrier status, and level of CR.

## Materials and Methods

### Study Design and Participant Selection

**FIGURE 1:**
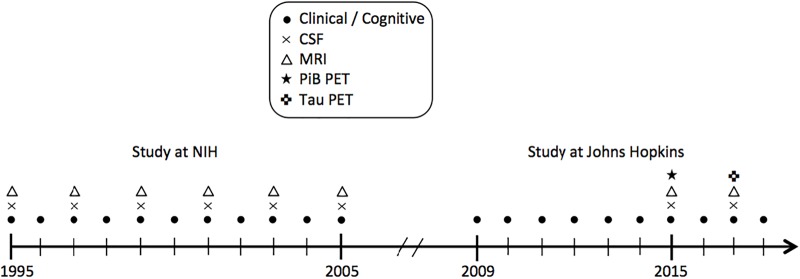
Timeline showing the design of the BIOCARD study, and types of data collected each year from 1995 to 2018. CSF indicates cerebrospinal fluid; MRI, magnetic resonance imaging; NIH, National Institutes of Health; PET, positron emission tomography; PiB, Pittsburgh compound B.

Data were derived from the BIOCARD study, an ongoing longitudinal prospective cohort study designed to identify variables among cognitively normal individuals that predict subsequent development of mild to moderate symptoms of AD. The study was initiated in 1995 at the National Institutes of Health (NIH). At baseline, following a comprehensive evaluation, 349 cognitively normal individuals were enrolled after providing written informed consent. By design, approximately 75% of the cohort had a first degree relative with dementia of the Alzheimer type. Clinical and cognitive assessments were conducted annually and CSF and MRI scans were acquired every other year. The study was stopped in 2005 for administrative reasons, and re-established in 2009 by a team from Johns Hopkins University (JHU). In 2015, the collection of CSF and MRI scans was re-initiated and amyloid imaging was begun (see Figure 1 for a study timeline). This study was approved by the JHU Institutional Review Board. Details pertaining to participant recruitment, clinical evaluation, and cognitive assessments have been described elsewhere (Albert et al., 2014).

The present report examines cross-sectional data from 149 non-demented participants with NPTX2 measures, including 130 who also had rs-fMRI measures. The CSF and MRI scans were obtained on the same day or 1 day apart from one another for all participants, except for one participant with a 30-day gap. All CSF and MRI measures were collected between 2015 and 2017. Data collection in this cohort is ongoing.

### Cognitive and Clinical Assessments

Throughout the course of the study, clinical assessments, cognitive testing, and medical evaluations have been completed annually. The annual visits included a comprehensive neuropsychological battery and a semi-structured interview, based on the Clinical Dementia Rating (CDR) (Morris) (for details, see Albert et al., 2014). Using confirmatory factor analysis, scores from 12 of the tests in the neuropsychological battery were used to create 4 composite scores, reflecting verbal episodic memory, executive function, visuospatial processing, and language. For details about the confirmatory factor analysis, and calculation of the composite scores, see Supplemental Materials.

Consensus diagnoses in each participant have been completed by the staff of the JHU BIOCARD Clinical Core. All cases were handled in a manner comparable with those employed in the National Institute on Aging Alzheimer’s Disease Centers program. First a syndromic diagnosis was established, using three sources of information: (1) clinical data pertaining to the medical, neurological, and psychiatric status of the individual; (2) reports of changes in cognition by the individual and by collateral sources; and (3) decline in cognitive performance, based on review of longitudinal testing from multiple cognitive domains (and comparison to published norms).

Second, if a subject was deemed to be impaired, the decision about the likely etiology of the syndrome was based on the medical, neurologic, and psychiatric information collected at each visit, as well as medical records obtained from the subject, where necessary. More than one etiology could be endorsed for each subject (e.g., AD and vascular disease). The consensus diagnosis procedures followed the diagnostic recommendations incorporated in the NIA/AA working group reports for the diagnosis of MCI (Albert et al., 2011) and dementia due to AD (McKhann et al., 2011). The diagnosis of Impaired Not MCI was also a potential option, and typically reflected contrasting information from the CDR interview and the cognitive test scores (i.e., the subject or collateral source expressed concerns about cognitive changes in daily life but the cognitive testing did not show changes, or vice versa). Participants with a diagnosis of Impaired not MCI (*N* = 23) were included in the group of cognitively normal participants, but results were comparable when they were excluded from analysis. Diagnoses (and determination of likely etiology) were made without knowledge of the biomarker measures.

### Cognitive Reserve Composite Score

CR was assessed by a composite score based on three measures collected at study baseline (i.e., between 1995 and 2005): (1) scores on the National Adult Reading Test (Nelson, 1982); (2) scores on the Wechsler Adult Intelligence Scale-Revised vocabulary subtest (Wechsler, 1981); and (3) years of education. To calculate the composite, which served as a proxy for CR, these measures were z-transformed and then averaged.

### APOE Genotyping and Coding

APOE genotypes were determined by restriction endonuclease digestion of polymerase chain reaction amplified genomic DNA (performed by Athena Diagnostics, Worcester, MA). APOE ε4 carrier status was coded by an indicator variable, with ε4 carriers coded as 1 if they had at least one ε4 allele and non-carriers coded as 0.

### Cerebrospinal Fluid Assessments

Cerebrospinal fluid was collected via lumbar puncture after overnight fasting. NPTX2 was measured using enzyme-linked immunosorbent assay (ELISA). Standards for the ELISA include multiple wells of different known concentrations of purified recombinant NPTX2 protein that are used to establish the range of linearity and determine concentrations of NPTX2 in patient samples. Subject samples were run in duplicate and blind to diagnosis and plates. Currently the NPTX2 ELISA shows <2% variation between wells in the same plate (see Xiao et al., 2017 for additional details regarding the assay).

### Magnetic Resonance Imaging Acquisition and Preprocessing

The MRI scans examined here were obtained between 2015 and 2017 on a 3T MR system (Philips Healthcare, Best, The Netherlands). Participants received instructions to not move, keep their eyes closed, and relax their mind while inside the scanner. Resting state blood-oxygenation-level-dependent (BOLD) data were collected using an echo-planar imaging (EPI) sequence with the following parameters: number of slices = 48; field of view (FOV) = 212 × 212 mm^2^; voxel size = 3.3 × 3.3 × 3.3 mm^3^; time repetition (TR) / time echo (TE) = 3000/30 ms; flip angle = 75°. The duration of each scan session was 420 s and comprised of 140 functional volumes. Magnetization-prepared rapid gradient echo (MPRAGE) scans were also obtained and used for anatomical reference and image registration (TR = 6.8 ms, TE = 3.1 ms, shot interval 3000 ms, flip angle = 8°, FOV = 240 × 256 mm^2^, 170 slices with 1 × 1 × 1.2 mm^3^ voxels, and scan duration = 5 min 59 s).

The BOLD data underwent standard pre-processing steps (using SPM and in-house MATLAB scripts), including slice timing correction, realignment, normalization to Montreal Neurologic Institute (MNI) 152 volumetric space via MPRAGE image, spatial smoothing using a Gaussian filter with a full-width half-maximum of 4 mm (Hou et al., 2019). The BOLD image series were detrended and bandpass-filtered to 0.01 – 0.1 Hz to retain the low-frequency fluctuation components. To reduce the motion effect on functional connectivity, the preprocessed BOLD data underwent a scrubbing process in which image volumes manifesting a displacement of ≥0.5 mm relative the prior frame were discarded (Power et al., 2014). In addition, the frames acquired immediately prior and immediately after displaced frames were also discarded to account for temporal spread of artifactual signal resulting from the temporal filtering in the low-frequency functional signal (Chan et al., 2014). After motion scrubbing, there were 11 participants with <70 frames of remaining data. Results remained the same when these participants were excluded from analysis.

The MPRAGE images were used for brain volume quantification using an automatic processing tool, MRICloud (Mori et al., 2016) (^1^Johns Hopkins University, Baltimore, MD). Total cerebral cortex volume, corrected for total intracranial volume (using the ratio method), was used as a measure of atrophy.

### Construction of Functional Connectivity Networks

The motion scrubbed preprocessed BOLD data were parcellated into 114 region-of-interests (ROIs) estimated in MNI 152 volumetric space and grouped into 7 resting-state functional connectivity networks based on the parcellation by Yeo et al. (2011), which was derived by clustering regions with similar connectivity profiles using data from 1000 subjects (Yeo et al., 2011). Following parcellation, we calculated cross-correlation coefficients between all pairs of ROIs after factoring out nuisance covariates including whole brain signal, white matter signal, CSF signal, and six rigid-body head motion corrections. The correlation coefficients were then converted to z-scores using a Fisher-z transform and formed a 114 × 114 matrix. To quantify network-wise functional connectivity, the connectivity matrix was reduced from 114 × 114 to 7 × 7 by averaging the z-transformed values belonging to the same network. For the present analyses, we focused on the 5 cognitive networks: executive control network, default mode network, dorsal attention network, limbic network, and combined salience/ventral attention network (The visual network and somato-motor network were not examined as these regions tend to be less impacted by aging and AD).

### Statistical Analysis

Group differences in descriptive statistics were compared with two-tailed *t*-tests for continuous variables and with chi-square or Fisher’s exact test, as appropriate, for dichotomous variables, uncorrected for multiple comparisons.

The association between NPTX2 and diagnostic status was assessed with logistic regression, with diagnosis (cognitively normal = 0; MCI = 1) as the dependent variable, and NPTX2, age, sex, race, and years of education as predictors. The association between NPTX2 and functional connectivity was evaluated using linear regression, separately for each of the five rsfMRI networks, with connectivity scores as the dependent variable and NPTX2, age, sex, race, diagnostic status, and years of education as predictors. Sensitivity analyses were performed to determine whether degree of cerebral atrophy accounted for associations observed with rsfMRI networks.

To test whether CR, APOE-e4 status, or age modify the association between NPTX2 and resting-state connectivity or diagnostic status, the CR composite score, APOE-e4 status, and three interaction terms (cross-products) were added as predictors to the model: (1) CR × NPTX2, (2) APOE-e4 status × NPTX2, and (3) age × NPTX2. For significant interactions, *post hoc* analyses were performed to determine the direction of the associations. Models with the CR composite score did not adjust for education, as education is part of the CR composite score. A significance level of *p* < 0.05 was adopted for all analyses, which were run in SAS version 9.4. All continuous variables were standardized before model fitting.

## Results

Characteristics of participants in the analyses, stratified by diagnostic status, are shown in Table 1. Participants with MCI had lower MMSE scores, lower CR composite scores, lower levels of NPTX2, and were less likely to be Caucasian.

**Table 1:** Participant characteristics at baseline, stratified by diagnosis.

	Participants with NPTX2 data		Participants with NPTX2 and rs-fMRI data	
Variable	Cognitively normal (*N* = 130)	MCI (*N* = 19)	Cognitively normal (*N* = 113)	MCI (*N* = 17)
Age, mean number of years (SD)	69.2 (8.7)	72.0 (7.7)	68.8 (8.7)	71.6 (8.0)
Sex, number of females (%)	84 (64.6%)	11 (57.9%)	72 (63.7%)	11 (64.7%)
Race, number Caucasians (%)	128 (98.5%)	16 (84.2%)^∗^	111 (98.2%)	14 (82.4%)^∗^
APOE ε4 carriers, number (%)	46 (35.4%)	7 (36.8%)	43 (38.1%)	6 (35.3%)
Education, mean years (SD)	17.3 (2.2)	16.6 (2.9)	17.3 (2.2)	16.2 (2.8)
Education, range (min – max)	12 – 20	12 – 20	12 – 20	12 – 20
MMSE score, mean (SD)	29.3 (0.9)	27.4 (1.7)^∗∗∗^	29.3 (0.9)	27.5 (1.6)^∗∗∗^
CR composite score	0.2 (0.7)	−0.5 (1.0)^∗∗^	0.2 (0.7)	−0.7 (1.0)^∗∗^
Executive function composite	0.7 (1.1)	−1.1 (1.6)^∗∗∗^	0.7 (1.2)	−1.1 (1.7)^∗∗∗^
Episodic memory composite	1.7 (1.5)	−0.3 (1.6)^∗∗∗^	1.6 (1.4)	−0.2 (1.7)^∗∗∗^
CSF NPTX2	742 (437)	521 (290)^∗∗^	737 (447)	485 (240)^∗∗^
Connectivity – Default	–	–	0.14 (0.07)	0.13 (0.05)
Connectivity – Limbic	–	–	0.40 (0.19)	0.41 (0.25)
Connectivity – Dorsal attention	–	–	0.14 (0.08)	0.15 (0.09)
Connectivity – Salience	–	–	0.16 (0.06)	0.14 (0.03)
Connectivity – Executive control	–	–	0.12 (0.06)	0.11 (0.04)

*Significant differences between the group of cognitively normal and MCI participants are indicated as follows: ^∗^p < 0.05, ^∗∗^p < 0.01, ^∗∗∗^p < 0.005.*

### Relationship Between NPTX2 and Diagnostic Status

Results from the logistic regression analysis demonstrated that lower levels of NPTX2 were associated higher odds of a diagnosis of MCI [*odds ratio (OR)* = 2.85, *95% confidence interval (CI)* = 1.19 – 6.87, *p* = 0.019]. The results from the logistic regression evaluating the interactions between NPTX2 and CR, APOE-e4 status and age, with respect to diagnostic status, are shown in Table 2. There was a significant interaction between NPTX2 and CR (*p* = 0.028), but not between NPTX2 and APOE-e4 or age. The analysis stratified by high vs. low CR composite scores (using the median, and excluding the non-significant interaction terms) showed that lower levels of NPTX2 were associated with higher likelihood of MCI among individuals with low CR scores (*OR* = 3.11, *95% CI* = 1.07–9.01, *p* = 0.037), but not among those with high CR scores (*OR* = 1.26, *95% CI* = 0.36–4.39, *p* = 0.72). When the analysis was stratified by level of NPTX2 (low vs. high, using the median), the association between higher CR composite scores and reduced likelihood of MCI was significant among participants with low NPTX2 (*OR* = 4.90, *95% CI* = 2.01–11.9, *p* = 0.0005), but not those with high NPTX2 (*p* = 0.13).

**Table 2:** Results of logistic regression analysis assessing the association between CSF levels of NPTX2 and likelihood of MCI in relation to CR, APOE-e4 status, and age.

Variable	Estimate	SE	*p*-value
Age	−0.953	0.446	0.033
Sex – Male	−1.390	0.746	0.064
Race – White	−3.697	1.524	0.015
APOE e4 – Carrier	1.482	1.701	0.384
CR composite score	1.303	0.410	0.002
NPTX2	0.174	0.485	0.705
NPTX2 × CR	−0.959	0.440	0.028
NPTX2 × APOE-e4	4.134	2.239	0.065
NPTX2 × Age	0.292	0.472	0.537

*MCI, Mild Cognitive Impairment; CR, cognitive reserve.*

### Relationship Between NPTX2 and Resting-State Functional Connectivity

**FIGURE 2:**
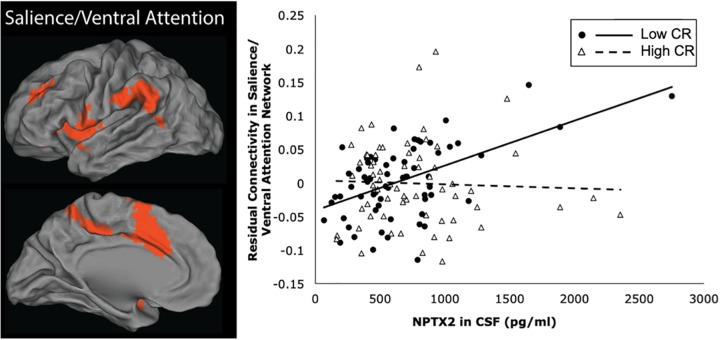
Brain regions within the salience/ventral attention network are shown in the left panel. The right panel shows a scatterplot of the partial correlation between residual functional connectivity in the salience/ventral attention network and levels of NPTX2 in CSF for individuals with high CR (filled circles, solid line) and lower CR (open triangles, dashed line), adjusted for age, sex, race, APOE-e4 genotype, and diagnostic status.

The results from the linear regression analyses examining the relationship between NPTX2 and functional connectivity are shown in Table 3. Higher levels of NPTX2 were associated with greater functional connectivity in the salience/ventral attention network (*p* = 0.012), but not with connectivity in any of the other networks (all *p* > 0.2). The association between NPTX2 and functional connectivity in the salience/ventral attention network remained significant when participants with MCI were excluded (*estimate* = 0.21, *SE* = 0.09, *p* = 0.025), as well as when participants with a diagnosis of Impaired not MCI (*n* = 20) were also excluded (*n* = 93, *estimate* = 0.22, *SE* = 0.09, *p* = 0.025). Additionally, older age was associated with reduced connectivity in the salience/ventral attention network, dorsal attention network, and default-mode network (all *p* ≤ 0.003), but connectivity did not differ by diagnostic status in any network. See Figure 2, left panel, for a graphical representation of the salience network, as defined in the current study.

**Table 3:** Results of linear regression analyses assessing the association between CSF levels of NPTX2 and resting-state functional connectivity in 5 networks.

Variable	Estimate	SE	*p*-value
Default mode	0.044	0.088	0.614
Limbic	0.046	0.089	0.604
Salience/ventral attention	0.219	0.085	0.012
Dorsal attention	−0.012	0.087	0.885
Executive control	0.105	0.090	0.249

*All analyses were adjusted for age, sex, race, years of education, and diagnostic status.*

Next we examined whether the association between NPTX2 and functional connectivity in the salience/ventral attention network was moderated by age, level of CR, or APOE-e4 genotype. As shown in Table 4, there were significant interactions between NPTX2 and age (*p* = 0.024) as well as between NPTX2 and CR (*p* = 0.017), indicating a stronger association between NPTX2 levels and functional connectivity among younger individuals and among individuals with lower CR composite scores, respectively. There was no significant interaction with APOE genetic status. Follow-up analyses stratified by CR composite scores (using the median) indicated that lower NPTX2 levels were related to reduced functional connectivity among individuals with low CR (*estimate* = 1.82, *SE* = 0.67, *p* = 0.009), but not among individuals with high CR (*estimate* = −0.28, *SE* = 1.59, *p* = 0.86). See Figure 2, right panel for scatterplot of the relationship between NPTX2 and connectivity in the salience/ventral attention network for individuals with high and low CR. The main effect of NPTX2, and its interaction with CR, remained significant when excluding participants with a diagnosis of MCI and Impaired not MCI, all *p* < 0.05.

**Table 4:** Results of linear regression analysis assessing the association between CSF levels of NPTX2 and resting-state functional connectivity in the salience/ventral attention network.

Variable	Estimate	SE	*p*-value
Age	0.038	0.163	0.817
Sex – Male	0.052	0.175	0.767
Race – White	−0.670	0.440	0.131
APOE e4 – Carrier	−0.298	0.326	0.363
CR composite score	0.315	0.170	0.067
Diagnostic status	0.216	0.293	0.463
NPTX2	1.803	0.711	0.013
NPTX2 × CR	−0.383	0.159	0.017
NPTX2 × APOE-e4	0.089	0.178	0.620
NPTX2 × Age	−1.617	0.706	0.024

*CSF, cerebrospinal; CR, cognitive reserve.*

*Post hoc* analyses were performed to examine the interactions with NPTX2 further. When stratifying by NPTX2 levels (high vs. low, using the median), the association between age and functional connectivity was stronger among those with high NPTX2 levels (*estimate* = −0.42, *SE* = 0.15, *p* = 0.007) compared to low NPTX2 levels (*estimate* = −0.16, *SE* = 0.10, *p* = 0.12). However, the CR composite score was not directly associated with functional connectivity in the salience/ventral attention network, whether levels of NPTX2 were high or low (both *p* > 0.5). Interactions between NPTX2 and CR, age, and APOE-genotype for other networks were not examined because they were not associated with NPTX2 levels in the primary analyses. Results remained similar when we additionally adjusted for individual differences in brain atrophy (total cerebral cortex volume).

Lastly, we ran an exploratory analysis to determine if functional connectivity in the salience/ventral attention network was associated with cognitive performance, hypothesizing that it might be related to executive functioning and possibly verbal episodic memory. Higher functional connectivity in the salience/ventral attention network was associated with higher executive function composite scores (adjusting for age, sex, race, APOE-e4 status, diagnosis, and CR composite scores, *estimate* = 0.21, *SE* = 0.10, *p* = 0.032), but not with the verbal episodic memory composite (*p* > 0.8; associations with visual-spatial processing and language were not examined, as there were no apriori hypotheses regarding these cognitive domains).

## Discussion

This study investigated the relationship between CSF levels of NPTX2, a synaptic protein that regulates synaptic plasticity and rhythmicity in cortical circuits, and resting-state functional connectivity in 5 intrinsically organized large-scale brain networks among a group of older individuals consisting of participants with normal cognition and subjects with MCI. Our findings confirmed previous reports of lower levels of CSF NPTX2 in subjects with MCI compared to controls (Xiao et al., 2017). Importantly, we found that higher levels of NPTX2 are associated with greater functional connectivity in the salience/ventral-attention network, but not in any other cognitive network. This association was independent of cerebral atrophy among the total sample, as well as the subgroup of individuals with normal cognition.

The salience/ventral attention network includes the mid to anterior cingulate gyrus, the insula, the inferior parietal lobule, and the inferior frontal gyrus. Activity in this network has been associated with the detection and selection of salient and behaviorally relevant exogenous and endogenous stimuli, response selection, and response inhibition (for reviews and meta-analyses, see Menon, 2015; Peters et al., 2016; Zhang et al., 2017). Recent work further indicates that the salience network acts as a dynamic switch between the default-mode network and the executive control network (Goulden et al., 2014; Chand and Dhamala, 2016). That is, the salience/ventral attention network appears to up-regulate the executive control network, allowing individuals to attend to task-relevant goals and simultaneously inhibit the default-mode network to suppress task-irrelevant, internally directed thought.

Since, NPTX2 is expressed primarily by pyramidal neurons in the cortex and hippocampus (O’Brien et al., 1999; Xiao et al., 2017); it is therefore unclear why the salience/ventral attention network might uniquely correlate with CSF NPTX2 levels. One potential possibility for the fact that decrements in NPTX2 were specifically related to lower connectivity in the salience/ventral attention network is that this network is more sensitive to aberrations in the balance of excitatory and inhibitory signaling compared to other networks due to its primary role in switching between the default mode and executive control networks. In line with this hypothesis, this switching function of the salience network has been shown to be impaired among individuals with executive MCI compared to cognitively normal older individuals (Chand et al., 2017).

Of note, prior studies are consistent with a mechanistic role for NPTX2 in fMRI measures of connectivity in cognitively normal older individuals. NPTX2 mediates adaptive strengthening of feed-forward and feedback inhibition onto pyramidal neurons by interneurons (Chang et al., 2010; Pelkey et al., 2015; Xiao et al., 2017), which are essential for gamma rhythmicity and synchrony (Buzsaki and Wang, 2012; Stroud and Vogels, 2018). However, the association between NPTX2 and gamma rhythmicity seems to require a primary insult that changes brain activity (Pelkey et al., 2015). For example, slice recordings from genetically modified adult mice lacking NPTX2 show near normal gamma oscillation frequency and power; however, in combination with a transgene that generates amyloidosis, these mice have a profound reduction of gamma power (Xiao et al., 2017). Thus, NPTX2 may be necessary to adapt to the effects of increases in the accumulation of abnormal proteins, such as amyloid-beta 1-42, or to other unknown perturbations that accrue with aging. Consistent with this hypothesis, a *post hoc* analysis of the data presented here demonstrated a stronger association between NPTX2 and rsfMRI connectivity in the salience/ventral attention network among cognitively normal individuals with more abnormal (i.e., lower) levels of CSF amyloid-beta 1-42 than those with more normal levels (data not shown).

To our knowledge, only two studies have investigated the relationship between NPTX2 and AD pathology in humans, and the results have not been entirely consistent. Higher NPTX2 was associated with higher CSF levels of both total tau (t-tau, a marker of general neurodegeneration) and phosphorylated tau (p-tau, a marker of neurofibrillary tau tangles) within both cognitively normal and AD-dementia patients, but not with CSF abeta 1-42 levels (Xiao et al., 2017). However, when combining across diagnostic groups (normal, MCI, and dementia), the association between NPTX2 and tau/ p-tau was negative and the association with abeta 1-42 was positive (both unadjusted for diagnostic status, Swanson et al., 2016). The association between NPTX2 and amyloid or tau as measured by brain imaging has yet been examined, highlighting the need for further investigations of this topic.

The results from this study may also provide some insights into the brain mechanisms associated with cognitive resilience and reserve. Prior studies suggest that NPTX2 may be a “resilience factor,”, based on its ability to balance excitation/inhibition (Chang et al., 2010; Pelkey et al., 2015) and the observed preservation of NPTX2 levels in brains of individuals who were cognitively normal at death but had neuropathological evidence of AD (sometimes referred to as asymptomatic AD) (Xiao et al., 2017). If NPTX2 confers resilience, one would predict that risk factors for AD, such as older age, APOE-e4 genotype, and level of CR would be more strongly associated with cognitive impairment or risk of progression to MCI among individuals with low levels of NPTX2. Although the number of participants with MCI was small in the current study and we only had cross-sectional data, our results provide some preliminary support for this idea, because lower CR scores were associated with greater likelihood of MCI only among participants with low NPTX levels. Future studies with larger samples of participants and AD biomarkers are needed to confirm the role of NPTX2 in providing resilience to cognitive impairment in the face of pathology.

The finding of an interaction between CR and NPTX2 with respect to both diagnostic status and functional connectivity in the salience/ventral attention network is also broadly consistent with prior longitudinal studies that have documented interactions between CR and markers of neuronal injury in relationship to the risk of progression to MCI. For example, the association between atrophy on MRI in selected AD-vulnerable regions and risk of progression to MCI appears to be greater among individuals with low CR than high CR (e.g., Soldan et al., 2015; Pettigrew et al., 2017). Future longitudinal studies are needed to examine interactions between NPTX2 and CR in order to confirm this hypothesis.

It is also noteworthy that greater functional connectivity in the salience/ventral attention network was related to higher scores on an executive function composite measure. These results strengthen prior findings indicating that the salience/ventral attention network is essential for modulation of attention and response selection (e.g., He et al., 2014; Li et al., 2017; Reineberg et al., 2018). Thus, low levels of NPTX2 may selectively affect executive functioning by modulating functional connectivity in the salience/ventral attention network.

The results from the current study must be interpreted within the context of its limitations. Participants are well educated, primarily Caucasian, and have a strong family history of AD-dementia. The results may therefore not generalize to the United States population at large. Additionally, the number of participants with MCI was small, which may explain why, unlike prior studies (see Badhwar et al., 2017), we did not find a difference in resting state functional connectivity between participants with normal cognition and those with MCI. The findings regarding the relationship of CSF NPTX2 and the salience/ventral attention network were, nevertheless, strong. It will be important to replicate the current results in larger, more diverse samples.

These findings emphasize the importance of examining NPTX2 in longitudinal CSF samples. For example, it is not yet known if NPTX2 measured in cognitively normal individuals predicts progression to MCI alone, or in combination with traditional AD biomarkers. The longitudinal relationship between NPTX2 and changes in functional connectivity also remain to be explored. Because CSF collection requires an invasive lumbar puncture, this may limit the potential broad application of NPTX2 measures. To overcome this limitation, efforts are underway to develop measures in blood based on neuronal-derived exosomes (Goetzl et al., 2018). These novel blood-based markers specifically focus on NPTX2 derived from neuronal sources because NPTX2 is also expressed in peripheral organs (Uhlen et al., 2015). Ultimately, such measures may be useful for identifying individuals at risk of cognitive decline, particularly in combination with traditional markers of AD pathology.

## Data Availability

This study data for the analyses presented in this report are available upon request from any qualified investigator for purposes of replicating the results.

## Ethics Statement

This study was carried out in accordance with the recommendations of the Johns Hopkins Institutional Review Board. All subjects gave written informed consent in accordance with the Declaration of Helsinki. The protocol was approved by the Johns Hopkins Institutional Review Board.

## Author Contributions

AS, KW, MA, and PW participated in study concept or design. XH, HL, AA, and MM collected and analyzed the MRI data. AM, M-FX, DX, and PW collected and analyzed the CSF data. AS performed the statistical analyses. AS, KW, CP, MA, and PW interpreted the data. AS, KW, CP, MA, and PW drafted and revised the manuscript for content. AM, XH, HL, MM, AA, DX, and M-FX reviewed and revised the manuscript for content.

## Conflict of Interest Statement

AS, AM, KW, CP, XH, HL, AA, and MM has ownership in Anatomy Works, LLC, a relationship which is being managed by the Johns Hopkins University. MA is an advisor to Eli Lilly. DX is a co-founder of CogNext. M-FX is a co-founder of CogNext. PW is a co-founder of CogNext.

## Footnotes

1 www.MRICloud.org
